# Populism and Feminism: Odd Bedfellows

**DOI:** 10.1007/s12115-017-0206-x

**Published:** 2017-12-18

**Authors:** Rob Kroes

**Affiliations:** Amsterdam, The Netherlands

**Keywords:** American contemporary politics, Populism, Feminism, Representative democracy, Electoral systems

## Abstract

In this era of populist insurgency breaking the mold of democratic politics, two movements clashed.They represented opposite sides of the political spectrum, one emancipatory, the other exclusionary. One may be identified as feminism, the other as populism. This essay analyzes both concepts and explores their connection.

At one point, it may have appeared as almost a course of historical justice, a foreordained order of innings. America’s first black president would, after a full two terms of service, leave the stage bowing out to America’s first woman-president, thus bringing two cycles of emancipation to completion. It was not to be. In a bitter end-game the woman candidate, long ahead in the race, lost to a man who was the very antithesis of everything she stood for. It looked like feminism stepping up to misogyny and getting a drubbing. Emancipatory enthusiasm proved no match for populist rage.

Or did it? After all, the election victory was narrow. Trump had lost the popular vote by close to three million, and won the electoral college by a mere 77,000 votes decisively cast in three mid-Western swing states.^1^ The electoral college, originally set up as one further hurdle on the way to mob rule, and essentially anti-majoritarian in intention, now had produced the opposite effect and allowed the angry voice of an insurgent minority to drown out all others. The year 2016 may live on in our collective memory as having produced two fluke outcomes of electoral systems: Trump in the U.S., Brexit in the U.K. Britain, in the grip of a populist insurgency against the constraints imposed by its membership of the European Union, had opted to break the mold of its system of parliamentary democracy and instead to use the referendum, every populist’s chosen tool for the unmediated expression of the people’s voice. Plebiscitary autocracies thrive on them. In Britain, manipulated the people’s vote may have been, driven more by gut feeling and anger than by anything resembling rational reflection, yet the feeling was that “the people” had spoken. No matter that Britain’s system of representative parliamentarism as a rule does not open the floodgates for people directly to vent their pent-up anger, the political establishment instantly closed ranks and made the outcome of the referendum its own. The people had spoken. How ironic, then, that in the U.S. the people had spoken as well and had given a clear majority of their votes to the Democratic candidate, Hillary Clinton. Donald Trump could only claim victory due to the American system of counting the electoral vote, state by state, in a winner-take-all system, rather than the popular vote as it had been cast nation-wide. It is a system that in fact resembles the standard British way of counting votes, district-by-district. Both systems make it hard for minority voices to get a fair hearing. If the Founding Fathers had set up the Electoral College as a further hurdle to stem the people’s direct expression of their collective will, it failed to do exactly that in the 2016 presidential election. In fact, Trump had promised a “Brexit-style victory,” and in many ways, it was. Only not in the ways electoral systems had worked out, neither in Britain, nor in the United States.

So, fluke or not, the winner produced by the American electoral system was, without a doubt - without “hanging chads,” so to speak - Donald Trump. On the day following his inauguration, Washington D.C. saw women marching in their hundreds of thousands, easily beating attendance numbers for Trump’s inauguration. The organizers of the march must have had another prognosis in mind, anticipating along with so many political pollsters and pundits a Clinton victory. It was not to be. Had women flocked with equal enthusiasm to the voting booths on November 8th, 2016, they would collectively have burst through another glass ceiling and put the first woman-president in American history in the White House. As one newspaper reminded the reader, on the day of the March: “American women voted overwhelmingly for Clinton, except the white ones.” Of college-educated white women a mere 51% had voted for Clinton. Of the non-college-educated a full 62% had preferred Trump. Yet there they marched, conjuring a view of women’s power a day after the inauguration of Donald Trump, serial adulterer and philanderer, misogynist, male chauvinist, vulgarian-in-chief. Visually back to back, on two consecutive days on the Washington Mall, what story do the photographic images of citizens gathered on the Mall, main forum of American democratic freedom, tell us? Was this a confrontation between populism and feminism? And if so, had this clash of worlds been in the cards all along, are feminism and populism irreconcilable movements, or had there been a parting of ways at some point in history?

This is how Hillary Clinton, in the first interview she gave since her election defeat, addressed the subject.^2^ “Certainly misogyny played a role” in her loss, she said. “That just has to be admitted.” Clinton acknowledged that Democrats need to do a better job reaching working-class Americans. She added that part of her problem reaching out to a population group coping with economic hardship, consisted in “layering on the first woman president over that, and I think some people, women included, had real problems.” This may not be the most trenchant analysis of Clinton’s weaknesses as a presidential candidate. Misogyny played its role, but it is far from accounting for the high number of white women supporting the Trump camp.

The election may have seemed like a slam-dunk for feminism, yet obviously on election day other issues weighed in, shifting the political balance away from the feminist agenda. And along with feminism a range of related emancipatory agendas, foremost to do with race and ethnicity, sank below the surface. Narrowly victorious, a brand of populism burst forth onto the political stage, following in the footsteps of a pied piper as if mesmerized. As Timothy Garton Ash put it, in a piece in the Guardian: “Every day one wakes up and thinks, ‘How on earth can this trashy mountebank be president of the United States?’ It is the character of the man which is the fundamental problem here, more than his ideology and policies, to the extent that one can detect any coherence in them.”^3^ There is no coherence or consistency, other than the impulse to deconstruct and demolish the institutions set up by preceding administrations in their pursuit of domestic and international policy goals, more particularly as they are connected to the name of his immediate predecessor Barack Obama. It is possible to see Trump’s every act in the Oval Office, signing executive orders, repealing regulations and rules issued by his immediate predecessor, as a sop to groups composing his populist constituency such as white nationalists and nativists, the Christian right, protectionist business interests, Tea Party insurgents resisting taxes and a black man in the White House. Trump’s moves to restrict abortion, defund Planned Parenthood, permit discrimination against LGBT people in the name of “religious liberty” and allow churches to become active in politics by gutting the Johnson Amendment (meant precisely to prohibit such activity) along with his nominations of judges championed by the conservative Federalist Society and his call for a ban on Muslim immigrants, all this has endeared him to nativists, the Alt-Right and the Christian right. He has rolled back civil rights legislation and business and environmental regulations. He has elevated several stalwarts of the Christian right into power—Mike Pence to the vice presidency, Jeff Sessions to the Justice Department, Neil Gorsuch to the Supreme Court, Betsy DeVos to the Department of Education, Tom Price to Health and Human Services and Ben Carson to Housing and Urban Development. He embraces the white supremacy, bigotry, American chauvinism, greed, religious intolerance, anger and racism that define his supporters.

As a measure of his success and an indication of his constituency, Trump had 81% of white evangelicals behind him. In the quid pro quo of political exchange there is a clear balance here. Yet where is the interest of white women in all this? There is no sign of lip service on Trump’s part to anything to do with women’s interests, to any item of a feminist agenda. Are they simply subsumed under one or other of the various labels comprising Trump’s populist support, gullibly taken in by one or another of his empty promises, to bring back jobs, to keep out Mexicans (“rapists, criminals”), to bring back an America of affluence, nostalgically remembered if not re-imagined, in short: to make America great again? Are they among the duped celebrants of what one might call a contemporary cargo cult, with Trump descending from on high in his magnificent silver bird, the name Trump grandly displayed on its sides?

If so, it would powerfully illustrate the appeal of contemporary populism and its promise of a reconstituted “people,” of a nation ridding itself of alien and un-American elements. Populism, in this view, is a homogenizing, anti-pluralist political force rising in defense of a narrow reading of what constitutes the nation. It is an angry insurgency against representative democracy seen as skewed and rigged against the real nation. In that sense, it stands in direct opposition to the democratizing enthusiasms most recently seen in the 1960s. Contemporary populism is still frantically engaged in the continuing effort to roll-back the gains and accomplishments of the emancipatory, and interconnected movements, as they emerged at the time. They spread out from the early civil-rights and student movements to include a wide range of demands for equal rights and political participation, the feminist agenda foremost among them. Like populism these emancipation movements also presuppose the existence of representative democracy. Only in their case they are out to widen and open up the system, not to narrow it down; they are aiming at a system of representation that is more inclusive, not more restrictive, in the access it offers to the citizenry.^4^

If this is one way to put populism and feminism at contradictory ends of the political spectrum and to see them as mutually exclusive, aren’t we being too contemporary in our use of populism as a negative concept, as a dark force in the workings of democracies? Are we doing justice to the long history of populism in American history and to the enthusiasms it inspired? In 1906 the German sociologist (and socialist) Werner Sombart raised the question of why there is no socialism in the United States. Given that America, like Germany, had gone through a tempestuous period of industrialization and, like Germany, was a relative late-comer to industrial and urban modernity – why was there no similar rise of a militant workers’ movement? His essay has become one of the main exhibits in the case for America being an exception in the history of Western nations, a case commonly referred to as American exceptionalism. But how truly exceptional was America’s experience? Had Sombart looked beyond America’s cities he would have struck upon a vast canvass of political ferment and protest, related to the impact of industrialization: the farmers’ protest in the mid-West. It took aim at banking and railroad interests, which although directly affecting farming life in the mid-West had concentrated their commanding heights in the big East-Coast cities. Like industrial workers in the cities farmers organized their collective voice and power, in a farmers’ movement infused with a sense of being “the people” – with all the entitlement bestowed by America’s hallowed texts, its Declaration of Independence, its Bill of Rights, Lincoln’s Gettysburg Address - rising against “the interests.” Politically they organized in a party known as the People’s Party. Their wider movement was commonly referred to as “populism.” For sure, in honesty to Sombart’s view, crucial elements were missing in American farmers’ populism to warrant calling it a form of socialism. First among these was the element of ideology. Calling their movement socialist, conceiving of it in the logic of a socialist ideology, would have been alien to their world view. Marx’s dictum that a group’s social position defines its collective outlook and understanding of itself – its ideology – never led mid-Western farmers to turn critics of capitalism. Yet in terms of the matrix of conflict – of productive labor set against the combined exploitative forces of financiers and railroad interests, Marx would have recognized the outline of class conflict.^5^

So, what we have here is clearly a political movement claiming access to the representative democratic system, finding its voice among other voices, adding their demands to the political agenda. Their struggle was one among many, adding to the pluralism of the democratic arena. Their use of the word “people” to describe their constituency was never exclusionary, their sense of the interests that inspired their actions could transcend barriers of race and forge interracial political alliances. In all these respects, early American populism finds its place alongside so many other emancipatory groups vying for access to America’s democratic politics.

Yet, at about the same time, political movements manifested themselves, which never called themselves populist, yet show striking similarities to what a moment ago we described as contemporary populism, anti-pluralist, exclusionary, narrowing down the sense of who constitute the people. We need only think of the anti-immigration movement, centering on ideas of racial purity, Anglo-Saxonism, One Hundred Percent Americanism, the re-emergence of the Klan, to get the picture. These were purely populist movements in the negative sense described above.

Yet, like the cat in the old American folk song, populism in its positive sense kept coming back. As almost a matter of cycles in American history – to use Arthur Schlesinger’s felicitous metaphor – populism again inspired democratic enthusiasms in the years of the Great Depression. As a positive term, in praise of the farmer, if not the yeoman farmer, seen as personifying the central character of the American republic, the 1930s rang with language celebrating the “common man.” As a theme in American historiography, populism as an inspirational social movement found its rediscovery, if not its full recovery, in a classic study by J. D. Hicks, published in 1931. As one reviewer, Roscoe D. Martin, put it: “In these days of ‘hard times,’ when unemployment and agricultural surpluses combine to make the lot of the workingman seem hard, when the leaders of bygone days arise once more to champion the cause of the people … the memory of students of American history, economics and politics goes back forty years to the agrarian uprising at the end of the last century.”^6^ There they are, the central inspirational words, “the cause of the people.” They would resound in political discourse, and find reflection in the popular culture of the time, haltingly and hesitantly at first. Among the songs that deal seriously with the Depression and that have some relevance to memories of populism are "Brother, Can You Spare a Dime?" (1932) by E. Y. Harburg and Jay Gorney and “Remember My Forgotten Man” (1933) by Al Dubin and Harry Warren. The songs share some interesting similarities. First, both songs depict the disenfranchised individual, forgotten and left behind. Both feature structures that contrast a driving, martial rhythm and melody in one section with a more lyrical section. Both are written in a minor key, which adds an element of poignancy. But as America struggled to leave misery and depression behind it, its culture picked up these signs of hope. The tone became more celebratory, singing the praise of the common man, as in Aaron Copland’s *Fanfare for the Common Man* or his *Lincoln Portrait*, both using a vernacular musical idiom to facilitate reception and resonance, or in the remarkable popular front-inspired *Ballad for Americans*, an operatic folk cantata as it has been called, made famous by black baritone singer Paul Robeson.^7^ The ballad sang the praise of the American people as it had been formed through the tribulations of its history from the American Revolution through the industrial revolution. It dramatically slows at the entrance of Robeson’s repeated line, “And you know who I am,” which over the course of the ten-minute song is questioned regularly (“No. Who are you, mister?”) In response, Robeson proudly announces that he is the “nobody who’s everybody”: the mechanics, musicians, teachers, and farmers whose belief in the nation is informed by their identities as Negro, Russian, Czech as well as Jewish, Methodist, and atheist. This diversity of perspectives is not a liability in “Ballad”; it is instead the exceptional contribution and wealth of this nation’s citizens, suggesting that the work of the nation is ongoing, but more perfect with each arrival. If the song spoke in the voice of an America in all its multiple facets, it tellingly managed to find a hearing among groups of equally wide range. Thus, In the 1940 presidential campaign it was sung at both the Republican National Convention (by baritone Ray Middleton) and that of the Communist Party.^8^

How distant all this sounds today, when we have a government of the “one percent for the one percent”, parading as a populist regime that pretends to see it as its mission to protect and defend all those people that have been jettisoned and left behind by the great transformative processes of globalization. It is a populism that no longer shows its American colors. There is nothing of the inclusiveness, of the encompassing embrace of “the nation” in all its variety and diversity, not for racial minorities, not for immigrants, not for women, nor any of the emancipatory action groups vying for access to the democratic forum of American politics. The wreckers’ ball of the nihilists now in power in the White House has seen to that. It has demolished the agenda of feminism along with the larger agenda of Hillary Clinton and the Democratic Party, in much the same way that it has set out to destroy the house that Obama built.

Rob Kroes is professor emeritus and former chair of the American Studies program at the University of Amsterdam, where he taught until September 2006. He is Honorary Professor of American Studies at the University of Utrecht and is a past president of the European Association for American Studies (EAAS, 1992-1996). He is the founding editor of two series published in Amsterdam: *Amsterdam Monographs in American Studies* and *European Contributions to American Studies*.

## Footnotes

1 To put Trump’s electoral college vote in perspective, we should point out that Trump’s electoral strength was concentrated in the American heartland, disparagingly referred to as fly-over America. As a measure of how well Trump did in the heartland, he won 2626 counties in the United States. Hillary Clinton won only 487. To put things in greater perspective, though, we should also point out the following: Of 136.6 million votes officially counted, Hillary Clinton got 65.8 million and Trump 62.9 million. So yes, Hillary beat Trump by 2.9 million votes, yet in fact Trump lost the *total popular vote* by 10.7 million. After all, Trump and Clinton were not the only candidates for the Presidency.

2
https://www.nytimes.com/2017/04/08/opinion/sunday/hillary-clinton-free-to-speak-her-mind.html?_r=0

3
https://www.theguardian.com/commentisfree/2017/jul/28/trump-brexit-end-of-west

4 For a discussion of populism along similar lines, see: Jan-Werner Müller, *What is Populism?* (Philadelphia: University of Pennsylvania Press, 2016) Also, Cas Mudde, “The Problem with Populism” (*The Guardian,* 17 February, 2015; https://www.theguardian.com/commentisfree/2015/feb/17/problem-populism-syriza-podemos-dark-side-europe)

5 Christopher Lasch, in his 1969 *The Agony of the American Left*, points to ideology as the element critically missing from the history of America’s protest movements, giving an original twist to Sombart’s older views. Particularly the chapter on “the decline of populism” elaborates the theme that the absence of an ideological framework prevented populism from developing a fuller critique of capitalist society.

6 As quoted by Martin Ridge in *Reviews in American History*, 1985.

7 Shana L. Redmond, “‘And you know who I am’: Paul Robeson sings America.” (*The Massachusetts Review*)

8 “Ballad for Americans” (1939), originally titled “The Ballad for Uncle Sam”, is an American patriotic cantata with lyrics by John La Touche and music by Earl Robinson. It was written for the Federal Theatre Project production “Sing for Your Supper” that opened on April 24, 1939.

